# Characterization of the effects of cannabinoid receptor deletion on energy metabolism in female C57BL mice

**DOI:** 10.3389/fendo.2024.1386230

**Published:** 2024-06-19

**Authors:** Morgan Sotzen, Ahmed Ahmed, L. Karl Olson, Omayma Alshaarawy

**Affiliations:** ^1^ Department of Family Medicine, Michigan State University, East Lansing, MI, United States; ^2^ Department of Physiology, Michigan State University, East Lansing, MI, United States

**Keywords:** cannabinoids, female, body weight, glucose metabolism, thermogenesis

## Abstract

**Background:**

Despite the evidence that energy balance is regulated differently in females and that the endocannabinoid system is sexually dimorphic, previous studies on the endocannabinoid system and energy balance predominantly used male models. Here, we characterize the effects of cannabinoid receptor deletion on body weight gain and glucose metabolism in female C57BL mice.

**Methods:**

Female mice lacking the cannabinoid-1 receptor (CB1R^-/-^), cannabinoid-2 receptor (CB2R^-/-^), or both receptors (CB1R^-/-^/CB2R^-/-^) and wild-type (WT) mice were fed with a low (LFD; 10% of calories from fat) or high-fat diet (HFD; 45% of calories from fat) for six weeks.

**Results:**

Female WT mice fed with HFD gained significantly more weight than WT mice fed with LFD (p < 0.001). Similar pattern was observed for CB2^/-^ mice fed with HFD compared to CB2R^-/-^ mice fed with LFD (p < 0.001), but not for CB1R^-/-^ fed with HFD vs. LFD (p = 0.22) or CB1R^-/-^/CB2R^-/-^ fed with HFD vs. LFD (p = 0.96). Comparing the 4 groups on LFD, weight gain of CB1R^-/-^ mice was greater than all other genotypes (p < 0.05). When fed with HFD, the deletion of CB1R alone in females did not attenuate weight gain compared to WT mice (p = 0.72). Female CB1R^-/-^/CB2R^-/-^ mice gained less weight than WT mice when fed with HFD (p = 0.007) despite similar food intake and locomotor activity, potentially owing to enhanced thermogenesis in the white adipose tissue. No significant difference in weight gain was observed for female CB2R^-/-^ and WT mice on LFD or HFD. Fasting glucose, however, was higher in CB2R^-/-^ mice fed with LFD than all other groups (p < 0.05).

**Conclusion:**

The effects of cannabinoid receptor deletion on glucose metabolism in female mice were similar to previously published findings on male mice, yet the effects on body weight gain and thermogenesis were attenuated in CB1R^-/-^ mice.

## Introduction

In 2021, more than 52 million people (18.7%) aged 12 years and older in the United States (US) used cannabis at least once in the past 12 months (1). The most well-known bioactive compound that is derived from the *Cannabis* plant, delta-9-tetrahydrocannabinol (THC), stimulates appetite and increases food intake (2). These actions are mediated mainly by the cannabinoid-1 receptors (CB1R), one of the most abundantly expressed G-protein coupled receptors in the brain (3). Furthermore, CB1R are located and functional in peripheral tissues that assist with energy balance including adipose and hepatic tissues (4). Early reports indicate that the pharmacological inactivation of CB1R decreases food intake and weight gain (5, 6). In these studies, the anorectic effects of CB1R antagonists were transient, whereas their effects on body weight were sustained owing to increased energy expenditure (7). In genetic knockout models, mice lacking CB1R (CB1R^-/-^) also displayed leaner phenotype and improved glucose clearance when compared to wild-type (WT) mice (8).

The other cannabinoid receptor, cannabinoid-2 (CB2R), shares an overall 44% amino acid similarity with CB1R and 68% similarity specific at the transmembrane areas (3). Although the two receptors share relatively similar structure, CB2R are found less frequently in the brain, and are expressed in several peripheral tissues, but predominantly in the immune system (9). Whereas CB1R play a major role in energy metabolism, the extent of CB2R influence on metabolic regulation is yet to be determined as the current evidence is inconclusive. The CB2R agonist, JWH-015, reduced food intake and body weight in obese mice (10). Conversely, the administration of a different CB2R agonist, JWH-133, promoted insulin resistance and fatty liver in mice fed with a high fat diet (HFD) (11). On the other hand, young mice lacking CB2R (CB2R^-/-^) had attenuated body weight gain and improved glucose clearance when fed with HFD with 60% of calories from fat (12). As CB2R^-/-^ mice aged, their food intake and body weight increased compared to WT mice (13). Furthermore, in our studies of mice lacking both CB1R and CB2R (CB1R^-/-^/CB2R^-/-^), we reported a lean phenotype and improved glucose clearance compared to WT mice (14). The resistance of CB1R^-/-^/CB2R^-/-^ to diet-induced obesity was likely mediated via CB1R as we did not observe a similar phenotype in mice lacking CB2R only (14).

Previous mouse studies investigating the role of the cannabinoid receptors in energy metabolism predominantly utilized male models, despite the evidence that energy balance is regulated differently in females (15). Additionally, sexual dimorphism has been demonstrated in several actions of the endocannabinoid system, including appetite regulation (16, 17). For example, male guinea pigs were more sensitive to the effects of pharmacological regulation of CB1R activity on food intake than females (18). Characterizing the effects of cannabinoid receptor modulation on energy metabolism in females is crucial given the therapeutic potential of the endocannabinoid system in metabolic disorders and that more US females experience severe obesity than males (11.5% vs. 6.9%) (19, 20). Herein, we hypothesized that the effects of cannabinoid receptor modulation on energy metabolism to be attenuated in female mice, similar to guinea pigs. Specifically, we used female mice lacking CB1R, CB2R or lacking both CB1R and CB2R to evaluate the contribution of the cannabinoid receptors in regulating body weight and glucose metabolism.

## Methods

### Animals

Mice deficient in CB1R were kindly provided by G. Kunos at the National Institutes of Health (Bethesda, MD, USA) (21). To develop these mice, the CB1R gene was mutated in MPI2 embryonic stem cells and homozygous mice were bred by back-crossing to C57BL mice (22). Mice deficient in CB2R originally created by Deltagen (Deltagen Inc. CA, USA) were obtained from Jackson Laboratory (strain:005786; Jackson Laboratory, ME, USA) (23). Mice deficient in both CB1R and CB2R were generated by breeding homozygous CB1R^-/-^ and CB2R^-/-^ mice. All mice were on C57BL background, housed and bred at Michigan State University and genotyped using Transnetyx (Cordova, TN). Seven-week-old WT mice were purchased from Jackson (strain: 000664; Jackson Laboratory, ME, USA) and acclimated for 1 week prior to diet administration.

### Housing

Mice were housed with up to 5 animals per cage. Rooms were maintained at 21 to 24°C and 40 to 60% humidity with a twelve-hour light-dark cycle. All procedures were performed in accordance with guidelines set forth by Michigan State University Institutional Animal Care and Use Committee, and the United States of America regulations concerning the use of animals in research.

### Body composition, diet, and body weight

At baseline (prior to diet administration; eight-week-old), body fat mass was measured using a Time Domain-Nuclear Magnetic Resonance–based analyzer (Minispec LF50; Bruker, MA, USA). Mice were then fed *ad libitum* either a low-fat diet with 10% of calories from fat and 70% of calories from carbohydrates (LFD; D12450B, Research Diets, NJ, USA), or HFD with 45% of calories from fat and 35% of calories from carbohydrates (D12451, Research Diets, NJ, USA) and water. Body weight was recorded weekly for 6 weeks. At the end of the experiment, body composition analysis was repeated.

### Food intake and indirect calorimetry

A subset of mice were individually housed in metabolic cages (TSE PhenoMaster/LabMaster System, MO, USA) after diet acclimation for ~5 days to measure metabolic performance, activity, drinking, and feeding. Ambient temperature was maintained at 20 to 23°C throughout analysis and the airflow rate through the chambers was adjusted to maintain an oxygen differential of ∼0.4% at resting conditions. Food intake, oxygen consumption (VO2: the difference between oxygen input and output) and carbon dioxide production (VCO2: the difference between carbon dioxide output and input) in each chamber were monitored every 3-minutes, while locomotor activity (beam breaks in the x-, y-, and z-direction) was continuously recorded. Respiratory exchange ratio (RER) was measured as a ratio of VCO2 (ml/h/kg)/VO2 (ml/h/kg), and energy expenditure (heat production; kcal/Kg [body weight]/h) was measured as (CVO2 * VO2 + CVCO2 * VCO2)/1000. The default values for CVO2 and CVCO2 were 3.941 and 1.106, respectively. Data were continuously collected over 5 days, with the first 12 hours used for habituation but excluded from the analysis.

### Glucose tolerance test

After 6 weeks on diet, mice were fasted early in the morning for 5 hours. Fasting glucose levels were measured in tail vein blood using a Freestyle Lite Glucometer (Abbott, IL, USA). D-glucose solution (2 g/kg) was then administered to a subset of mice by intraperitoneal injection and blood glucose levels were measured at 30-, 60-, 90- and 120-minutes post-injection.

### Necropsy and tissue collection

Mice were deeply anaesthetized with isoflurane (3–5% for induction and 2–3% for maintenance). Blood samples were collected in heparinized tubes via cardiac puncture (Becton Dickinson, NJ, USA). Blood samples were centrifuged for 10 minutes at 2000 x g at 4°. Plasma was collected for measuring insulin levels using ultra-sensitive mouse insulin ELISA kit (Crystal Chem, IL, USA). Perigonadal white adipose tissue (WAT), scapular brown adipose tissue (BAT) and liver were snap frozen and stored at −80°C for further analyses.

### RNA analysis

Total RNA was obtained using TRIZOL (Invitrogen, CA, USA) and its concentration was determined by NanoDrop 2000 (Thermo Scientific, IL, USA). Total RNA was reverse transcribed into complementary DNA using High-Capacity cDNA Reverse Transcription Kit (Applied Biosystems, MA, USA). Real-time quantitative PCR (qPCR) was performed using SYBR Green Master Mix (Life Technologies, CA, USA), and mouse gene-specific primers (**Supplementary Table 1**). Relative amounts of mRNA were calculated using the comparative cycle threshold method and normalized to the abundance of ribosomal 18s RNA using the ABI PRISM Sequence Detection System (Applied Biosystems, MA, USA).

### Statistical analysis

Data are presented as mean ± standard error (SEM). Student’s t-test was used to compare LFD-fed mice with HFD-fed mice in the same genotype. One-way analysis of variance (ANOVA) was used to compare different genotypes on the same diet. When outcomes were repeatedly measured over time, repeated measures ANOVA was used to compare genotypes on the same diet. Tukey’s test for multiple comparisons was used to test all possible pairwise differences whenever ANOVA (one-way or repeated) was statistically significant at the 0.05 level. Analyses were performed using GraphPad Prism 7 (GraphPad Software, CA, USA).

## Results

### Body weight

Prior to diet administration, mean body weight of female WT mice was 18.5 ( ± 0.2) grams (gm). There were no differences in baseline body weight between WT mice and CB1R^-/-^ mice (18.1 ± 0.4 gm; *p =* 0.65). Female mice lacking CB2R (20.4 ± 0.3 gm) or lacking both cannabinoid receptors (20.0 ± 0.3 gm) mice were slightly heavier than both WT and CB1R^-/-^ mice at baseline (*p <*0.05). Body composition analysis revealed less fat mass for CB1R^-/-^ and CB1R^-/-^/CB2R^-/-^ mice at baseline compared to WT and CB2R^-/-^ mice (*p <*0.05).

**Figure 1:**
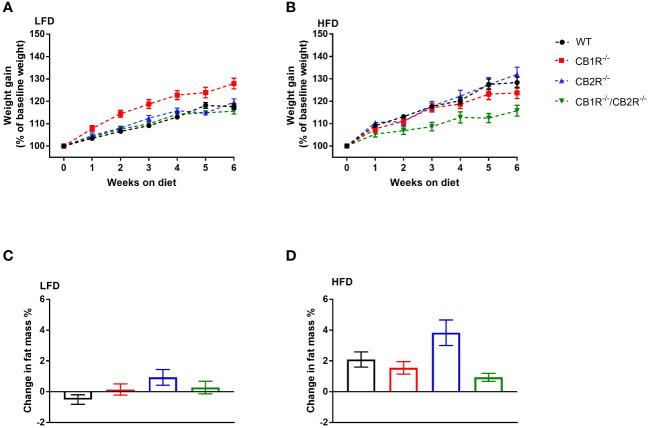
Body weight gain of female mice lacking the cannabinoid receptors Arrows indicate the comparison with WT mice. **(A)** Body weight gain of female mice on LFD for 6 weeks (n = 16-24/group). Repeated measure ANOVA F_genotype_ = 9.7; *p <*0.001. **(B)** Body weight gain of female mice on HFD for 6 weeks (n = 14-25/group). Repeated measure ANOVA F_genotype_ = 4.9; *p* = 0.004. **(C)** Change in body fat mass of female mice when fed with LFD for 6 weeks (n = 9-23/group). One-way ANOVA: F = 2.2, p = 0.09. **(D)** Change in body fat mass of female mice when fed with HFD for 6 weeks (n = 13-23/group). One-way ANOVA: F = 4.5, p = 0.006.

**Figure 2:**
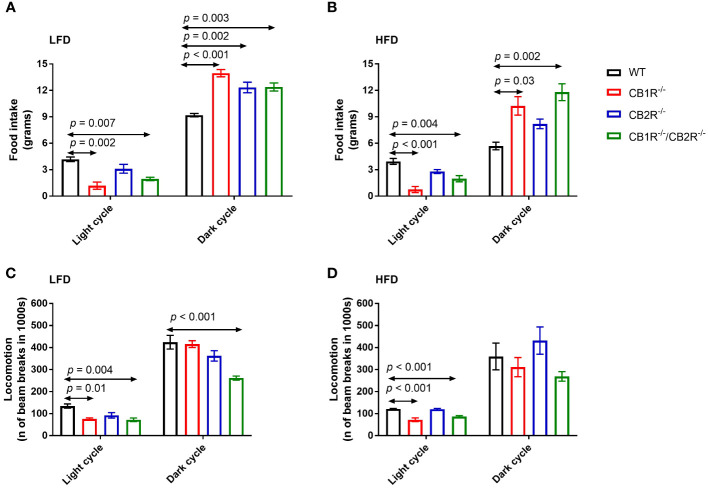
Food intake and locomotion of female mice lacking the cannabinoid receptors. Arrows indicate the comparison with WT mice. **(A)** Food intake of female mice for 108 hours on LFD (n = 3-6/group). One-way ANOVA for light cycle: F = 9.0, *p* = 0.001; for dark cycle: F = 13.8, *p* < 0.001. **(B)** Food intake of female mice for 108 hours on HFD (n = 4-6/group). One-way ANOVA for light cycle: F = 14.5, *p* < 0.001; for dark cycle: F = 8.1, *p* = 0.003. **(C)** Locomotion of female mice for 108 hours on LFD (n = 4-6/group). One-way ANOVA for light cycle: F = 6.7, *p* = 0.004; for dark cycle: F = 11.8, *p <*0.001. **(D)** Locomotion of female mice for 108 hours on HFD (n = 3-6/group). One-way ANOVA for light cycle: F = 23.3, *p* < 0.001; for dark cycle: F = 2.5, *p* = 0.10.

When fed with LFD with most calories from carbohydrates for 6 weeks, female WT mice gained an average of 3.3 ± 0.2 gm (weight gain = 17.6%). Weight gain of CB2R^-/-^ (3.9 ± 0.3 gm; 19.6%) and CB1R^-/-^/CB2R^-/-^ (3.2 ± 0.3 gm; 15.6%) was not different from WT mice (**Figure 1A**). Body weight gain of CB1R^-/-^ (4.6 ± 0.3 gm; 27.8%) was relatively greater than WT (*p* < 0.001), CB2R^-/-^ (*p* = 0.005) and CB1R^-/-^/CB2R^-/-^ mice (*p <*0.001). Yet, weight gain of female CB1R^-/-^ mice was not explained by changes in body fat mass as there were no robust differences observed between the 4 groups (**Figure 2C**).

Compared to LFD, body weight gain of female WT mice was greater when mice were fed with HFD with 45% of calories from fat (5.3 ± 0.5 gm; weight gain = 28.3%; *p* < 0.001). Similar pattern was observed for CB2R^-/-^ mice (weight gain on HFD = 6.8 ± 0.7 gm; 32.0%; *p* < 0.001), whereas weight gain of female CB1R^-/-^ (4.1 ± 0.4 gm; 23.5%; *p* = 0.22) and CB1R^-/-^/CB2R^-/-^ (2.9 ± 0.4 gm; 15.8%; *p* = 0.96) mice was similar when mice were fed with HFD compared to their LFD-fed littermates.

When comparing the 4 genotypes on HFD, CB1R^-/-^/CB2R^-/-^ mice (*p* = 0.007) gained less weight compared to WT mice, whereas weight gain of CB1R^-/-^ (*p* = 0.72) and CB2R^-/-^ (*p* = 0.99) was not different from WT mice (**Figure 1B**). Female CB2R^-/-^ mice gained more fat mass when fed with HFD compared to CB1R^-/-^ (*p* = 0.04) and CB1R^-/-^/CB2R^-/-^ (*p* = 0.005; **Figure 1D**). There were no differences in body fat change between WT, CB1R^-/-^ and CB1R^-/-^/CB2R^-/-^ mice (*p* > 0.05).

### Food intake, locomotion, RER and indirect calorimetry

All genotypes ate greater quantities of the LFD compared to their HFD-fed littermates (*p <*0.05), except for CB1R^-/-^/CB2R^-/-^ mice which ate similar amounts of food when fed with LFD or HFD (*p* = 0.6). Compared to WT mice, mice lacking the cannabinoid receptors ate relatively less during the light cycle and more during the dark cycle (**Figures 2A, B**). There were no statistically significant differences in food intake when comparing CB1R^-/-^ and CB1R^-/-^/CB2R^-/-^ mice, whereas CB2R^-/-^ mice ate more during the light cycle compared to CB1R^-/-^mice.

Female CB1R^-/-^ and CB1R^/-^/CB2R^-/-^ mice exhibited less locomotor activity during the light cycle compared to WT mice (**Figures 2C, D**). No differences in locomotion were between CB2R^-/-^ and WT mice (*p* > 0.05). Female CB1R^-/-^/CB2R^-/-^ exhibited less locomotor activity than all other groups, that was robust during the dark cycle when mice were fed with LFD. When fed with HFD, no significant differences in locomotion were observed between the 4 genotypes during the dark cycle (*p* > 0.05).

**Figure 3:**
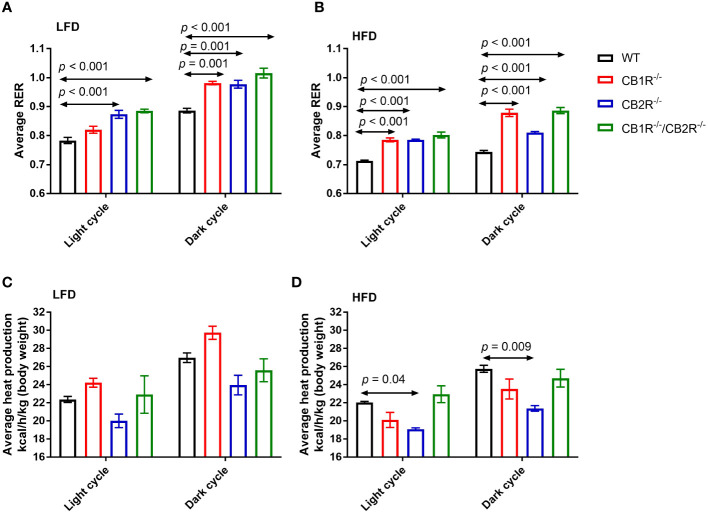
Respiratory exchange ratio (RER) and heat production of female mice lacking the cannabinoid receptors. Arrows indicate the comparison with WT mice. **(A)** RER of female mice for 108 hours on LFD (n = 4-6/group). One-way ANOVA for light cycle: F = 15.9, *p <*0.001; for dark cycle: F = 15.8, *p <*0.001. **(B)** RER of female mice for 108 hours on HFD (n = 4-6/group). One-way ANOVA for light cycle: F = 34.6, *p <*0.001; for dark cycle: F = 58.6, *p <*0.001. **(C)** Heat production of female mice for 108 hours on LFD (n = 4-6/group). One-way ANOVA for light cycle: F = 3.0, *p* = 0.06; for dark cycle: F = 6.2, *p* = 0.007. **(D)** Heat production of female mice for 108 hours on HFD (n = 4-6/group). One-way ANOVA for light cycle: F = 7.2, *p* = 0.004; for dark cycle: F = 5.7, *p* = 0.009.

Comparing all genotypes on LFD, RER was lower for WT mice than mice lacking the cannabinoid receptors (**Figure 3A**). Mice lacking CB1R exhibited lower RER than CB2R^-/-^ and CB1R^-/-^/CB2R^-/-^ mice during the light cycle (*p* < 0.05) whereas no significant differences in RER were observed for CB1R^-/-^, CB2R^-/-^ and CB1R^-/-^/CB2R^-/-^ during the dark cycle.

Consistent with the use of fat as substrate, RER was reduced for all groups when fed with HFD compared to their LFD-fed littermates (*p* < 0.05). Comparing all genotypes on HFD, RER was lower in WT mice than mice lacking the cannabinoid receptors (**Figure 3B**). RER was higher for CB1R^-/-^ and CB1R^-/-^/CB2R^-/-^ mice than CB2R^-/-^ mice during the dark cycle (*p* < 0.05). Finally, there were no statistically significant differences in energy expenditure when comparing CB1R^-/-^ and WT mice, whereas energy expenditure was relatively lower for CB2R^-/-^ mice than all other groups (**Figures 3C, D**).

### Glucose metabolism

**Figure 4:**
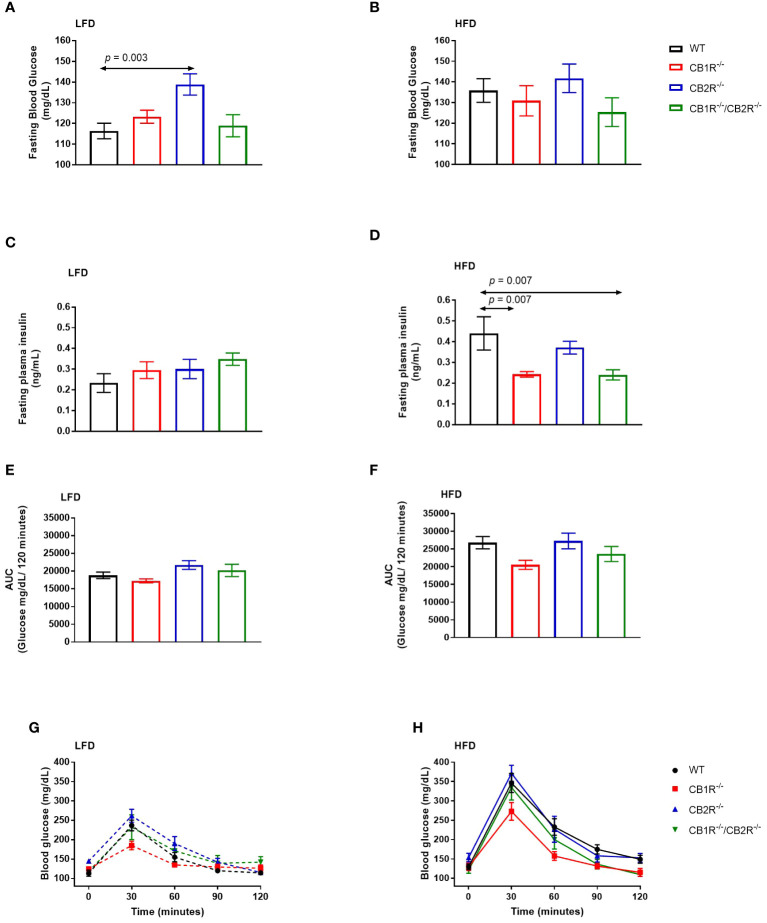
Glucose metabolism of female mice lacking the cannabinoid receptors Arrows indicate the comparison with WT mice. **(A)** Fasting blood glucose of female mice when fed with LFD for 6 weeks (n = 12-25/group). One way-ANOVA = 5.3; p = 0.002. **(B)** Fasting blood glucose of female mice when fed with HFD for 6 weeks (n = 9-19/group). One way-ANOVA = 0.9; p = 0.5 **(C)** Fasting blood insulin of female mice when fed with LFD for 6 weeks (n = 3-11/group). One way-ANOVA = 0.7; p = 0.6. **(D)** Fasting blood insulin of female mice when fed with HFD for 6 weeks (n = 5-8/group). One way-ANOVA = 6.7; p = 0.002 **(E, G)** Blood glucose levels of female LFD mice after intrapertonial injection of D-glucose solution (2 g/kg; n = 5-9). Area under the curve one way-ANOVA F = 3.8; p = 0.03. **(F, H)** Blood glucose levels of female HFD mice after intrapertonial injection of D-glucose solution (2 g/kg; n = 6-9). Area under the curve one way-ANOVA F = 3.0; p = 0.05.

When fed with LFD, fasting glucose of female CB1R^-/-^ mice (123.3 ± 3.1 mg/dL) was not significantly different from WT mice (116.4 ± 3.8 mg/dL; *p* = 0.60). Similarly, no differences were observed when comparing CB1R^-/-^/CB2R^-/-^ (118.9 ± 5.3 mg/dL; *p* = 0.98) and WT mice, whereas fasting glucose of female CB2R^-/-^ mice (138.9 ± 5.2 mg/dL) was higher than WT (*p* = 0.003), CB1R^-/-^ (*p* = 0.03) and CB1R^-/-^/CB2R^-/-^ mice (*p* = 0.02; **Figure 4A**).

When fed with HFD, mean fasting glucose of female WT mice (135.8 ± 5.7 mg/dL) was higher than their LFD-fed littermates (p = 0.01). No differences in fasting blood glucose were observed for CB1R^-/-^ mice (130.9 ± 7.3 mg/dL), CB2R^-/-^ mice (141.8 ± 6.9 mg/dL), or CB1R^-/-^/CB2R^-/-^ (125.3 ± 7.0 mg/dL) when fed with LFD or HFD (*p >*0.05). Comparing the 4 genotypes on HFD, there were no significant differences in fasting glucose levels (**Figure 4B**).

There were no differences in fasting insulin between the 4 genotypes when fed with LFD (**Figure 4C**). On the other hand, mean fasting insulin levels were lower for CB1R^-/-^ mice and CB1R^-/-^/CB2R^-/-^ mice than their WT mice (**Figure 4D**) when fed with HFD. Fasting insulin levels were also lower among CB1R^-/-^ (*p* = 0.07) and CB1R^-/-^/CB2R^-/-^ (*p* = 0.07) mice when compared to CB2R^-/-^ mice, but these differences failed to reach statistical significance. There were no differences in fasting insulin levels between CB2R^-/-^ and WT mice (*p* = 0.61).

When fed with LFD, there were no differences in glucose clearance between WT, CB1R^-/-^ and CB1R^-/-^/CB2R^-/-^ mice (*p* > 0.05; **Figures 4E, G**) when injected with D-glucose solution, whereas CB2R^-/-^ displayed worse glucose clearance when compared to CB1R^-/-^ mice (*p* = 0.02). When fed with HFD, glucose clearance was reduced in all genotypes when compared to their LFD-fed littermates (*p* < 0.05; data are not shown in figures), but there were no significant differences in glucose clearance when comparing the 4 genotypes on HFD (**Figures 4F, H**).

### Gene expression

#### Thermogenesis

**Figure 5:**
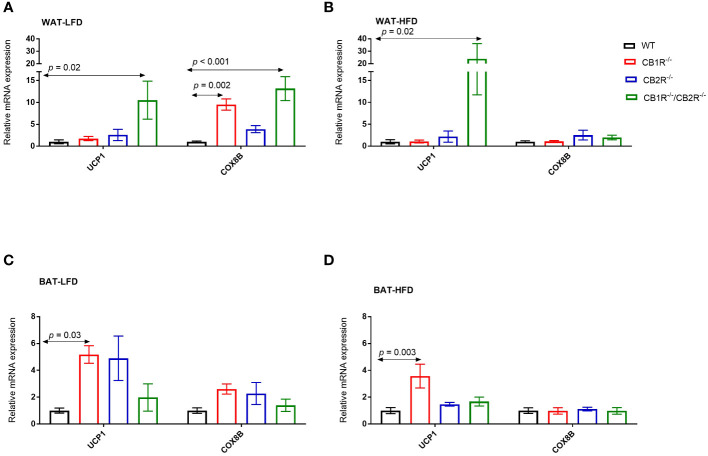
Expression of thermogensis genes in adipose tissue of female mice lacking the cannabinoid receptors Arrows indicate the comparison with WT mice. **(A)** Relative expression of UCP1 (One way-ANOVA = 4.4; *p* = 0.01; n = 7-13/group) and COX8B (One way-ANOVA = 11.2; *p* < 0.001; n = 7-17/group) in the WAT of female mice fed with LFD for 6 weeks. **(B)** Relative expression of UCP1 (One way-ANOVA = 5.0; *p* = 0.001; n = 6-13/group) and COX8B (One way-ANOVA = 0.9; *p* = 0.46; n = 7-14/group) in the WAT of female mice fed with HFD for 6 weeks. **(C)** Relative expression of UCP1 (One way-ANOVA = 3.9; *p* = 0.02; n = 6-16/group) and COX8B (One way-ANOVA = 2.1; *p* = 0.12; n = 5-16/group) in the BAT of female mice fed with LFD for 6 weeks. **(D)** Relative expression of UCP1 (One way-ANOVA = 5.4; *p* = 0.004; n = 8-11/group) and COX8B (One way-ANOVA = 0.1; *p* = 0.96; n = 8-11/group) in the BAT of female mice fed with HFD for 6 weeks.

In all genotypes, the expression of uncoupled protein 1 (UCP1) in the WAT was not different when females were fed with LFD or with HFD for 6 weeks (*p*> 0.05; data are not shown in figures). Comparing the 4 genotypes on LFD, the level of UCP1 mRNA was significantly higher in CB1R^-/-^/CB2R^-/-^ mice compared to all other groups (**Figure 5A**). No differences in UCP1 expression were found when comparing WT, CB1R^-/-^ or CB2R^-/-^ mice fed with LFD. Similar findings were observed when comparing UCP1 expression in mice fed with HFD (**Figure 5B**).

Similarly, the expression of UCP1 in the BAT was not different in all genotypes when female mice were fed with HFD compared to their LFD-fed littermates (*p*> 0.05). When comparing the 4 genotypes, the expression of UCP1 was higher in CB1R^-/-^ mice than WT mice when fed with LFD (*p* = 0.03; **Figure 5C**) or HFD (*p* = 0.003; **Figure 5D**). Also, levels of UCP1 mRNA were higher in CB1R^-/-^ mice than CB2R^-/-^ mice (*p* = 0.02) when fed with HFD. There were no statistically significant differences in UCP1 expression in the BAT when comparing WT, CB2R^-/-^ or CB1R^-/-^/CB2R^-/-^ mice.

Comparing the 4 genotypes on LFD, levels of cytochrome c oxidase subunit 8B (COX8B) mRNA were higher in CB1R^-/-^ and CB1R^-/-^/CB2R^-/-^ mice in the WAT compared to WT and CB2R^-/-^ mice (*p* < 0.05; **Figure 5A**). When fed with HFD, the expression of COX8B was upregulated in the WAT of WT mice (mean difference = 2.9; *p* = 0.01) and downregulated in CB1R^-/-^ mice (mean difference = -0.6; *p* = 0.001) compared to their LFD-fed littermates. The expression of COX8B was also higher in CB2R^-/-^ mice (mean difference = 1.5; *p* = 0.21) and lower in CB1R^-/-^/CB2R^-/-^ mice (mean difference = -0.4; *p* = 0.12) fed with HFD than their LFD-fed littermates, but these differences were not statistically significant. The expression of COX8B was similar in the 4 genotypes when female mice were fed with HFD (**Figure 5B**).

Like WAT, COX8B was downregulated in the BAT of CB1R^-/-^ mice fed with HFD when compared to CB1R^-/-^ mice fed with LFD (mean difference = -0.5; *p* = 0.01). The expression of COX8B was not different in the BAT when comparing the 4 genotypes on LFD (**Figure 5C**) or HFD (**Figure 5D**).

#### CB1R expression

In female WT mice, the expression of CB1R in the WAT, BAT and liver was not significantly different when mice were fed with LFD or with HFD for 6 weeks (*p*> 0.05; data are not shown in figures). Interestingly, the expression of CB1R decreased in the WAT of female CB2R^-/-^ (mean difference = -0.9; *p* = 0.01) when mice were fed with HFD compared to their LFD-fed littermates. A similar reduction that was not statistically significant was observed in the liver (*p* = 0.09).

**Figure 6:**
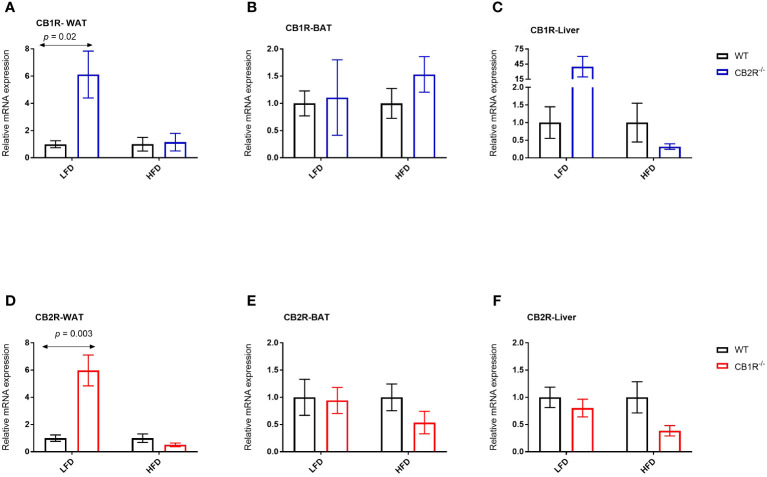
Expression of CB1R and CB2R genes in adipose tissue and liver of female mice Arrows indicate the comparison with WT mice. **(A)** Relative expression of CB1R in WAT (LFD n = 8-10/group; HFD n = 5-10/group) **(B)** Relative expression of CB1R in BAT (LFD n = 5-6/group; HFD n = 10-11/group) **(C)** Relative expression of CB1R in liver (LFD n = 8-13/group; HFD n = 7-13/group) **(D)** Relative expression of CB2R in WAT (LFD n = 8/group; HFD n = 8-11/group) **(E)** Relative expression of CB2R in BAT (LFD n = 9-13/group; HFD n = 6-10/group) **(F)** Relative expression of CB2R in liver (LFD n = 8-13/group; HFD n = 7-14/group).

Comparing WT and CB2R^-/-^ on LFD, the level of CB1R mRNA in the WAT and liver was significantly higher in CB2R^-/-^ mice compared to WT mice (**Figures 6A, C**). No statistically significant differences in CB1R expression were found in the BAT (**Figure 6B**) or when mice were fed with HFD (**Figures 6A–C**).

#### CB2R expression

The expression of CB2R increased when female WT mice were fed with HFD compared to their LFD-fed littermates in the WAT (mean difference = 19.2; *p* = 0.02) and liver (mean difference = 4.2; *p* = 0.03). Similarly, the expression of CB2R increased in the liver of female CB1R^-/-^ mice fed with HFD compared to their LFD-fed littermates (mean difference = 1.5; *p* = 0.03), whereas a non-significant increase was observed in the WAT (mean difference = 0.7; *p* = 0.18).

When fed with LFD, the level of CB2R mRNA in the WAT was significantly higher in CB1R^-/-^ mice compared to WT mice (**Figure 6D**). No differences in CB2R expression were found in the BAT, liver or when mice were fed with HFD (**Figures 6D–F**).

## Discussion

Despite the well-established sexual dimorphism of the endocannabinoid system, mouse models used for studying its role in energy regulation have been limited by the underrepresentation of female animals (16, 17). The goal of this study was to characterize the effects of cannabinoid receptor deletion on energy metabolism in female C57BL mice. The data presented here showed that the deletion of CB1R alone in female mice did not attenuate body weight gain, an effect that is consistently seen in male mice (5, 6). When fed with LFD (14% fat; 70% carbohydrates), mean body weight of male CB1R^-/-^ mice was reduced within 5 weeks of initiating diet compared to WT mice (8). Faster changes were observed when mice were fed with HFD (49% fat; 33% carbohydrates); mean body weight of male CB1R^-/-^ mice was reduced after 3 weeks of HFD compared to WT mice (8). In our studies of female mice, there were no differences in baseline body weight between WT mice and their CB1R^-/-^ littermates. Interestingly, weight gain of CB1R^-/-^ mice was relatively greater than WT mice when fed with the carbohydrate-rich LFD for 6 weeks, whereas their weight gain was not significantly different when fed with moderate-carbohydrate HFD. Diurnal differences in food intake were observed for female CB1R^-/-^ and WT mice but the 24-hr food intake was similar in both groups, in contrary to published finding on male mice (24, 25). Weight gain of female CB2R^−/−^ mice fed with LFD or HFD was not different from WT mice on the same diet, whereas female CB1R^-/-^/CB2R^-/-^ mice fed with HFD gained less weight compared to CB2R^−/−^ and WT mice, similar to males (14). Yet, the attenuated weight gain of CB1R^-/-^/CB2R^-/-^ mice was not explained by decreased food intake or increased locomotion.

There were no substantial differences in energy intake between the 4 genotypes. Total energy expenditure, on the other hand, includes resting metabolic rate, thermogenesis and physical activity. In agreement with prior evidence on male mice, deletion of CB1R, CB2R or both receptors in female mice was not associated with enhancement of the spontaneous locomotor activity (11, 14, 26, 27). Female WT and CB1R^−/−^ mice had similar body mass composition, whereas CB2R deletion increased fat mass when mice were fed with HFD. In agreement, CB2R stimulation via the selective agonist, JWH-015, reduced fat mass by 30-40% in obese male C57BL mice compared to vehicle (10). The increase in body fat mass observed in female CB2R^−/−^ mice can affect the resting metabolic rate of this genotype and potentially explain the reduced energy expenditure observed when mice were fed with HFD.

In previous studies of male mice, energy expenditure slightly increased in mice lacking CB1R compared to WT mice, potentially indicating enhanced thermogenesis owing to the lack of differences in spontaneous locomotor activity (27). To support this hypothesis, the administration of CB1R antagonists increased the expression of UCP1 in the BAT of male mice (28). Enhanced thermogenesis also modified adipocyte biology and promoted browning of the WAT in male mice lacking CB1R in the adipose tissue (29). These findings were attenuated in female mice; Energy expenditure was largely similar for female CB1R^-/-^ and WT mice and the expression of thermogenesis genes was not different in the WAT of both genotypes. The expression of UCP1 in the BAT, however, remained higher in female CB1R^-/-^ mice fed with LFD or HFD than WT mice fed with the same diet. Similar to CB1R^-/-^, energy expenditure was not different between female CB1R^-/-^/CB2R^-/-^ and WT mice (14). Yet, the increase in the expression of thermogenesis genes in the WAT, previously reported in male CB1R^-/-^/CB2R^-/-^ mice, was still observed in female mice (14).

Higher fasting glucose was observed in female CB2R^-/-^ when fed with LFD, whereas no differences in fasting glucose, insulin or glucose clearance were observed for CB1R^-/-^, CB1R^-/-^/CB2R^-/-^ and WT mice. Zibolka et al. studied glucose metabolism in male and female CB1R^-/-^ and CB2R^-/-^ mice fed with standard diet (30). They reported lower glucose levels in male CB1R^-/-^ mice compared to WT mice, whereas consistent with our findings, no differences were detected in female mice (30). Also, higher blood glucose levels were detected in both male and female CB2R^-/-^ mice compared to WT mice (30). In our study, the higher fasting blood glucose for CB2R^-/-^ mice fed with LFD was not replicated when mice were fed with HFD, suggesting that the detrimental effects of high fat feeding on circulating blood glucose levels in C57BL mice outweigh the contribution of CB2R deletion in glucose metabolism in female mice. In agreement and consistent with published male studies, there were no differences in fasting insulin levels or glucose clearance between female CB2R^-/-^ and WT mice fed with HFD (12). On the other hand, relatively lower fasting insulin and improved glucose clearance were detected in female CB1R^-/-^ mice fed with HFD compared to WT and CB2R^-/-^ mice, suggesting a stronger contribution of CB1R deletion in glucose metabolism (8, 14).

It is possible that the expression of one cannabinoid receptor is modified by the deletion of the other cannabinoid receptor, affecting the phenotype of the knockout model. Indeed, CB1R expression in the metabolically active-WAT and liver increased in female CB2R^-/-^ mice fed with LFD compared to WT mice fed with the same diet. The activation of CB1R has been linked to weight gain and impaired glucose metabolism in previous studies (31, 32). When fed with HFD, these differences were attenuated, potentially indicating a modulatory role of diet on the endocannabinoid system. Indeed, levels of circulating endocannabinoids increased in response to HFD in previous studies and this increase was associated with decrease in CB1R expression, potentially via a feed-back counter-regulatory mechanism (33, 34). This might explain the reduction of the ‘already’ elevated CB1R expression in female CB2R^-/-^ mice when fed with HFD, compared to LFD. Yet, we did not observe similar changes in female WT mice, suggesting that CB1R and the type of diet both contribute to the observed phenotype of female CB2R^-/-^ mice in our study.

On the other hand, the expression of CB2R increased in the WAT and liver when WT mice were fed with HFD compared to LFD, consistent with previous reports of marked induction of CB2R expression in male mice that correlated with WAT inflammation (11). The expression of CB2R in the WAT was significantly higher in CB1R^-/-^ mice compared to WT mice when fed with LFD, but only a minimal increase in CB2R expression was observed when female CB1R^-/-^ mice were fed with HFD compared to LFD. Overall, the expression of CB2R trended lower in CB1R^-/-^ mice than WT mice when fed with HFD, suggesting that like CB1R, CB2R and the type of diet modulate energy metabolism in female mice.

In summary, we characterized the effects of the cannabinoid receptor deletion on body weight gain and glucose metabolism in female mice. The effects of CB1R deletion on body weight gain and thermogenesis were attenuated in female mice, whereas the deletion of CB2R or CB1R^-/-^/CB2R^-/-^ were largely similar to published findings on male mice. Limitations of the study include the reliance on the widely published findings on male mice and the lack of direct comparison. All mice were on C57BL background, but it is possible that genetic drift develops over time and changes the phenotype of mice. Yet, the breeding scheme was limited to less than 10 generations and results from our study were consistent, regardless of the generation of the mice. Also, we did not control for estrus cycle phase in our study, which can introduce variability as mouse estrus cycle was found to influence CB1R in the brain (35). Future directions include understanding the effects of diet composition and sex hormones on the activity of the endocannabinoid system and its role in energy regulation.

## Data availability statement

The raw data supporting the conclusions of this article will be made available by the authors, without undue reservation.

## Ethics statement

The animal study was approved by Michigan State University Institutional Animal Care and Use Committee. The study was conducted in accordance with the local legislation and institutional requirements.

## Author contributions

MS: Writing – original draft, Methodology, Data curation. AA: Writing – review & editing, Methodology, Formal analysis, Data curation. LO: Writing – review & editing, Resources, Conceptualization. OA: Writing – review & editing, Supervision, Methodology, Funding acquisition, Formal Analysis, Conceptualization.
