# Supplementary material for: Characterization of the effects of cannabinoid receptor deletion on energy metabolism in female C57BL mice
Source: Front Endocrinol (Lausanne). 2024 Jun 19;15:1386230. doi: 10.3389/fendo.2024.1386230 (PMC11221337; doi:10.3389/fendo.2024.1386230)
Supplement: Supplementary file 1 [file Table_1.docx]

| **Table S1: PCR primer sequence** | | |
| --- | --- | --- |
| **Gene** | **Forward** | **Reverse** |
| **18s RNA** | GCAATTATTCCCCATGAACG | GGCCTCACTAAACCATCCAA |
| **UCP1** | GCATTCAGAGGCAAATCAGC | GCCACACCTCCAGTCATTAAG |
| **COX8B** | AGCCAAAACTCCCACTTCC | TCTCAGGGATGTGCAACTTC |
| **CBR1** | GTACCATCACCACAGACCTCCTC | GGATTCAGAATCATGAAGCACTCCA |
| **CBR2** | AGCTCGGATGCGGCTAGAC | AGGCTGTGGCCCATGAGA |
